# 3DFAACTS-SNP: using regulatory T cell-specific epigenomics data to uncover candidate mechanisms of type 1 diabetes (T1D) risk

**DOI:** 10.1186/s13072-022-00456-5

**Published:** 2022-06-30

**Authors:** Ning Liu, Timothy Sadlon, Ying Y. Wong, Stephen Pederson, James Breen, Simon C. Barry

**Affiliations:** 1 South Australian Health and Medical Research Institute, Adelaide, Australia; 2 Robinson Research Institute, University of Adelaide, Adelaide, Australia; 3 Bioinformatics Hub, School of Biological Sciences, Faculty of Health and Medical Sciences, University of Adelaide, Adelaide, Australia; 4 Women’s and Children’s Health Network, Women’s and Children’s Hospital, Adelaide, Australia; 5 Black Ochre Data Labs, Indigenous Genomics, Telethon Kids Institute, Adelaide, Australia; 6 John Curtin School of Medical Research, Australian National University, Canberra, Australia

**Keywords:** Type 1 diabetes, Autoimmune disease, Regulatory T cells, Hi-C, Transcription factor binding, Functional annotation

## Abstract

**Background:**

Genome-wide association studies (GWAS) have enabled the discovery of single nucleotide polymorphisms (SNPs) that are significantly associated with many autoimmune diseases including type 1 diabetes (T1D). However, many of the identified variants lie in non-coding regions, limiting the identification of mechanisms that contribute to autoimmune disease progression. To address this problem, we developed a variant filtering workflow called 3DFAACTS-SNP to link genetic variants to target genes in a cell-specific manner. Here, we use 3DFAACTS-SNP to identify candidate SNPs and target genes associated with the loss of immune tolerance in regulatory T cells (Treg) in T1D.

**Results:**

Using 3DFAACTS-SNP, we identified from a list of 1228 previously fine-mapped variants, 36 SNPs with plausible Treg-specific mechanisms of action. The integration of cell type-specific chromosome conformation capture data in 3DFAACTS-SNP identified 266 regulatory regions and 47 candidate target genes that interact with these variant-containing regions in Treg cells. We further demonstrated the utility of the workflow by applying it to three other SNP autoimmune datasets, identifying 16 Treg-centric candidate variants and 60 interacting genes. Finally, we demonstrate the broad utility of 3DFAACTS-SNP for functional annotation of all known common (> 10% allele frequency) variants from the Genome Aggregation Database (gnomAD). We identified 9376 candidate variants and 4968 candidate target genes, generating a list of potential sites for future T1D or other autoimmune disease research.

**Conclusions:**

We demonstrate that it is possible to further prioritise variants that contribute to T1D based on regulatory function, and illustrate the power of using cell type-specific multi-omics datasets to determine disease mechanisms. Our workflow can be customised to any cell type for which the individual datasets for functional annotation have been generated, giving broad applicability and utility.

**Supplementary Information:**

The online version contains supplementary material available at 10.1186/s13072-022-00456-5.

## Background

Autoimmune diseases are chronic inflammatory disorders caused by a breakdown of immunological tolerance to self-antigens, which results in an imbalance between multiple immune cells, including conventional T cells (Tconvs) and regulatory T cells (Tregs) [1]. The imbalance of immune cell function can lead to the destruction of host tissues, such as is observed in multiple autoimmune diseases, including rheumatoid arthritis (RA), multiple sclerosis (MS) and inflammatory bowel disease (IBD). In the case of type 1 diabetes (T1D), a reduction of Treg cell function contributes to unrestrained immune destruction of the insulin-generating pancreatic beta cells, resulting in the loss of control of blood sugar levels [2].

Regulatory T cell function is mediated by expression of the Foxhead Box Protein 3 (FOXP3) transcription factor (TF) as evidenced by severe autoimmune diseases observed in FOXP3-deficient scurfy mice [3] and IPEX in humans [4–6]. RNA sequencing and chromatin immunoprecipitation (ChIP) studies have uncovered an extensive FOXP3-dependent molecular program involved in Treg cell development and stability [7, 8]. Functional fitness of Treg is dependent on stable robust expression of FOXP3, such that reduced FOXP3 expression is linked to reduced Treg function. For example, in a small T1D cohort study, we have shown that there is a decrease in FOXP3 expression in the Treg of children over the first 9 months post diagnosis [9]. However, since FOXP3 itself is not mutated in autoimmune diseases other than IPEX, the loss of FOXP3 levels and functional fitness is likely caused by perturbation of the Treg gene regulatory network. Hence, by decoding the regulatory network of FOXP3, and mapping the genetic risk to the key functional genes it impacts, we will gain a better understanding of how autoimmune diseases like T1D could be countered.

T1D has a strong pattern of inheritance [10]. Genome-Wide Association Studies (GWAS) have identified over 50 loci that are strongly associated with T1D, based on the genotyping of a total of 9934 cases and 16,956 controls from multiple cohorts and resources [11]. In addition, fine-mapping of immune-disease associated loci represented on the Immunochip Array [12] followed by a Bayesian approach identified 44 significant T1D-associated Loci and over 1,000 credible SNPs [13]. Although GWAS and fine-mapping studies have revealed significant associations between genetic variants and T1D, the vast majority of the sampled single nucleotide polymorphisms (SNPs) are located in non-coding regions that do not alter the amino acid sequence in a protein, making it difficult to assign direct biological functions to variants [14–16]. Non-coding variants can be linked to direct changes in gene expression by identifying expression quantitative trait loci (eQTL) that aim to associate allelic changes to cis (within 1Mbp of the associated gene) and trans (> 1Mbp) changes in gene expression [17]. This additional direct gene expression association however still fails to identify direct mechanisms by which a specific genetic variant can change gene expression. In addition, usage of eQTLs to establish direct changes from GWAS variants is somewhat limited to local, or cis-eQTLs [18], whereas mounting evidence shows that long-range regulatory connections, driven by three-dimensional chromatin interactions [19, 20], can mediate these changes in expression.

With the increasing affordability and availability of high-throughput sequencing techniques and various epigenomics sequencing data protocols, the impact of genome organisation and accessibility can now be added to the functional annotation of genetic risk [1]. Chromatin immunoprecipitation sequencing (ChIP-seq) allows us to identify the binding sites of a transcription factor; assay for transposase-accessible chromatin sequencing (ATAC-seq) data offers the ability to identify accessible regions of the genome; and high resolution chromosome conformation capture sequencing (Hi-C) data can facilitate the investigation of the three-dimensional structure of the genome. Since it is believed that the mechanisms by which non-coding SNPs contribute to diseases are mostly via changes to the function of regulatory elements [16], we believe that combining multiple genomics and epigenetics sequencing data can further reveal the relationship between GWAS SNPs and disease pathways.

While alterations in either the effector or regulatory arms of the immune system can result in loss of tolerance and autoimmune disease, we have used a Treg-centric view of loss of tolerance. This is based on the observation that defects in Treg function have been reported in autoimmune diseases including T1D and MS [21, 22] and that experimental deletion of FOXP3 or reduced Treg function results in autoimmune disease in many model systems [23, 24]. Our hypothesis is that the genetic variation that specifically alters Treg function will reside in open chromatin in Treg cells that is bound by FOXP3 and the genes controlled by these regulatory regions can be identified by chromosome conformation capture approaches. Therefore, in this paper, we describe a filtering workflow using multiple sequencing datasets from human Tregs, aiming to identify plausible immunomodulatory mechanisms and potentially find previously unknown connections between causative variant SNPs significantly associated with T1D and the genes they impact. Only by doing this will a new wave of personalised medicines to prevent T1D be discovered.

## Results

### Post-GWAS filtering using Treg-specific epigenomic datasets prioritises functionally relevant genetic variants contributing to T1D

**Fig. 1:**
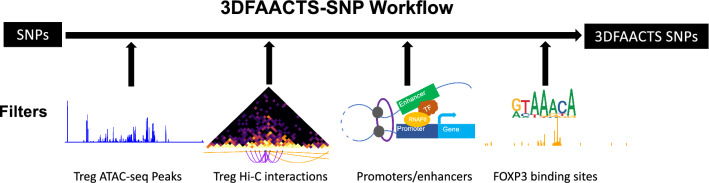
Diagram of the individual components of the Treg-specific 3DFAACTS-SNP filtering workflow for identifying variants that are potentially causative to type 1 diabetes (T1D). GWAS or fine-mapped variants (on the left) are intersected with different filtering elements, including Treg ATAC-seq peaks, interactions from Treg Hi-C, promoters or enhancers and previously identified FOXP3 binding regions in Treg cells [28], resulting in filtered variants we termed 3DFAACTS SNPs

As T1D is partly a consequence of Treg dysfunction, we infer that variants contained within active regulatory regions of Treg cells are likely to contribute to disease progression by impacting Treg function. A view supported by the finding that T1D-associated SNPs are enriched at Treg-specific regulatory regions [25]. Therefore, starting with published T1D GWAS variant information, we designed a filtering workflow (Fig. 1) using multiple human Treg-specific epigenomic data to identify perturbations within defined “regulatory T cell active regions”.

In order to obtain highly accessible chromatin regions in Treg, we performed transposase-accessible chromatin using sequencing (ATAC-seq) on resting and stimulated Treg cells from three donors and sequenced to an average of 37.1 million read pairs (± 4 million) per sample, with TSS enrichment score from 8.13 to 25.7 (Additional file 1: Fig. S1 and Table S1). From the ATAC-seq data, we identified ~ 577,866 ATAC-seq peaks on average, with over 100,000 and 200,000 peaks shared by the replicates in resting and stimulated Tregs, respectively (Additional file 1: Fig. S2). These ATAC-seq peaks were then merged into 683,954 non-redundant peaks and used to screen for variants located in accessible regions in regulatory T cells as the first filtering step of the 3DFAACTS-SNP pipeline (Fig. 1).

Numerous studies have shown that three-dimensional (3D) interactions play important roles in gene regulation, mediated by DNA looping bringing enhancers and promoters together at transcriptional hubs [26–28]. As a result, distant loci which physically interact with disease-associated regulatory regions can be potentially impacted by these regions. To identify 3D interaction in Treg cells, we generated and sequenced an in situ Hi-C library of Tregs to 1.3 billion read pairs and identified 345,259 statistically significant Hi-C interactions at 5-kb bin resolution after a series of stringent filtering processes (see “Methods”) (Additional file 1: Table S2). These Hi-C interactions were then used to connect ATAC-seq peaks containing disease-associated variants to other regions in Tregs.

To assign potential function to these variant associated ATAC-seq peaks and Hi-C interacting regions, we next determined the overlap of these regions using enhancer and promoter annotations. This included 113,369 enhancers (mean size of 698 bp) identified by the Functional Annotation of the Mammalian Genome (FANTOM5) project [29] and promoter regions (*n* = 73,171) associated with GRCh38/hg38 UCSC known transcripts. Promoters were defined by extending the sequence 2 kb upstream of transcription start sites (TSS). Additionally, we extended the list of regulatory regions using the 15-state chromHMM model for CD4 + CD25 + CD127− primary Treg cells from the Roadmap Epigenomics Project [30]. We defined chromHMM states *EnhG*, *Enh* and *EnhBiv* as enhancers and *TssA*, *TssAFlnk*, *TssBiv* and *BivFlnk* as promoters. FANTOM5 enhancers and defined promoters and chromHMM enhancers/promoters states were then merged, respectively, to represent all possible genetic regulatory elements, covering 7.49% of the genome (Additional file 2).

The transcription factor FOXP3 is critical for Treg function and orchestrating immunological tolerance, and stable high FOXP3 expression levels are observed specifically in Tregs [3, 31]. Therefore, by intersecting filtered SNPs with significant human FOXP3-binding signals, we can largely constrain SNPs within regulatory regions to FOXP3 controlled Treg-specific gene networks. In the pipeline, we used 8,304 FOXP3 ChIP-chip peaks (mean size = 1317 bp) from our previous study [31] to specify FOXP3 binding in human Tregs. Of interest, by searching the Gene and Autoimmune Disease Association Database (GAAD) [32], we obtained 245 annotated genes that are associated with T1D, and found a significant enrichment of FOXP3 binding sites in T1D-associated genes (Fisher exact test: *P*-value = 4.519e-09), suggesting a strong association between T1D risk and FOXP3 controlled Treg function. Taken together, FOXP3 binding, physical interaction, regulatory element and open chromatin regions offer a large subset of regions to use for GWAS variant prioritisation and functional annotation experiments.

### Linking fine-mapped T1D-associated variants to their targets via chromatin interactions

Genetic studies have identified over 50 candidate gene regions that contain potentially causative SNPs that impact T1D [11]. Recently, 1228 putative causal variants associated with T1D (99% credible set) were identified from a study by using Immunochip and a Bayesian fine-mapping method [13]. We used our workflow to further prioritise variants from this fine-mapped set to investigate potentially causative SNPs that contribute to T1D via affecting promoter/enhancer interaction in human Treg cells.

From the 1228 fine-mapped T1D-associated SNPs, we identified 36 variants that meet our filtering criteria: a variant must be in open chromatin region, where the chosen Hi-C interactions are contacting, overlapping with either a promoter or an enhancer region and bound by FOXP3 binding sties, in this study we will refer to them as T1D 3DFAACTS SNPs. To demonstrate the biological relevance of the process and the identified T1D 3DFAACTS SNPs, we performed 100 permutations with the workflow to confirm that the identification of 36 T1D-specific SNPs was significantly higher than by random chance (Fisher’s exact test, average *P*-value 7.22e-08) (Additional file 1: Fig. S3). These variants are located at 14 different chromosomal loci and distally interact with a further 266 regions three-dimensionally in Tregs (Table 1 and Additional file 3: Table S3). Most variants (71.4%, 25 out of 35 SNPs) were in enhancer regions rather than promoters while one variant, rs614120 is located in both the *TssAFlnk* chromHMM state and T cell-specific enhancers from FANTOM5. Given that a *TssAFlnk* state can either indicate a promoter or enhancer [33], combining with the identified FANTOM enhancer information, we believe that rs614120 is more likely to be located within an enhancer region.

**Table 1:** T1D 3DFAACTS SNPs identified using the 3DFAACTS-SNP filtering workflow from T1D fine-mapping SNPs

Chromosome	Position	SNP id	Nearest locus (linear distance)	Located within regulatory regions		Interacting genes (3D)
				Treg ChromHMM	FANTOM5 expressed enhancers	
chr2	204700689	rs12990970	CTLA4	TssAFlnk		**CD28,**CTLA4**,ICOS**
	204732714	rs231775		TssAFlnk		**RAPH1,CD28**,**ICOS**
	204738919	rs3087243		EnhG		**RAPH1**
chr3	46327588	rs11718385	CCR3	Enh		**XCR1,CCR2,CCR5AS,CCR5**
	46391390	rs6441972	CCR2	TssAFlnk		**CCR3**
	46401032	rs3138042		Enh		
	46411661	rs2856758	CCR5	Enh		**SLC6A20,FYCO1,CXCR6,XCR1, LOC105377067,CCR3,CCR1,CCR2**
	46412259	rs1799988		TssAFlnk		**SLC6A20**, **FYCO1**,**CXCR6, XCR1, LOC105377067,CCR3,CCR1,CCR2**
chr5	35852311	rs6890853	IL7R	TssAFlnk		**SPEF2,LOC105374724,CAPSL**
chr6	90948476	rs62408222	BACH2	Enh		BACH2,**LOC105377891**
	90983850	rs905671		Enh	✓	BACH2
	90984035	rs943689		Enh	✓	BACH2
	90995980	rs614120		TssAFlnk	✓	BACH2,**LOC105377891**
chr7	50462418	rs10216316	IKZF1	EnhG		**SPATA48**
	50462498	rs10215297		EnhG		**SPATA48**
	50465206	rs55981617		EnhG		**SPATA48,ZPBP,**IKZF1
	50465654	rs12670555		EnhG		**SPATA48,ZPBP,**IKZF1**,**
chr10	6088743	rs12722508	IL2RA	TssAFlnk		**RMB17**
	6094697	rs61839660		TssAFlnk		IL2RA,**RMB17,PFKFB3,LINC02649**
	6096667	rs12722496			✓	IL2RA,**RBM17**,**PFKFB3**,**LINC02649**
	6107534	rs11597367		Enh		IL2RA,**RBM17**
chr12	9910720	rs3176793	CD69	TssA		**LOC374443,CLEC2D**
	9912182	rs2160086		TssA		**LOC374443,CLEC2D**
	9912730	rs3176789		TssA		**LOC374443,CLEC2D,LOC105369728**
	9916640	rs3136559		Enh		**LOC374443,CLEC2D,LOC105369728**
	9925758	rs1029992		Enh		
	9926064	rs1029991		Enh		
	9926397	rs1029990		Enh	✓	
	9926624	rs10844749		Enh		
	9926784	rs1540356		Enh		
chr15	38903672	rs16967112	RASGRP1	Enh	✓	**FAM98B,**RASGRP1**,LOC105370775, LOC105370780,LINC02694,FSIP1**
	38903884	rs56249992		Enh		RASGRP1,**FAM98B, LOC105370775, LOC105370780,LINC02694,FSIP1**
chr16	11188949	rs71136618	CLEC16A	Enh		CLEC16A**,LOC107984859, LOC105371082,RMI2,SOCS1**
chr17	38755665	rs11656173	SMARCE1	Enh	✓	**CCR7**
chr18	12838767	rs17657058	PTPN2	Enh		**LINC01882,**PTPN2**,SEH1L**
chr22	30581722	rs5753037	HORMAD2	Enh		HORMAD2,**LIF-AS1,LIF**

The nearest locus indicates the closest gene to the variants in linear distance, while 3D interacting genes are genes in contact with the variants via Treg Hi-C interactions. Overlapped regulatory elements of each 3DFAACTS SNPs are displayed, including chromatin states from a 15-state model and expressed enhancers from FANTOM5. Detailed SNP and interaction information is contained in Additional file 3: Table S3

*Genes in bold indicate novel 3D interacting genes of the identified SNPs

Of the 14 loci identified, 8 contained more than two plausible variants across the loci. For example, variants located near the CD69 gene on chromosome 12 had the highest number of filtered variants, with 9 variants located in regulatory regions around the gene. In order to annotate the filtered variants to nearby genes, we took two approaches: annotated genes that were located in proximity to the SNPs using linear, chromosomal distances, and genes identified by their interaction with variant-containing regulatory regions via Treg Hi-C interactions (Table 1). Genes proximal to the identified 36 T1D variants include CTLA4, CCR5, IL7R, BACH2, IKZF1, IL2RA, CD69, RASGRP1, CCR3, CCR2, CLEC16A, HORMAD2 and PTPN2. These genes have previously been linked to T1D due to their proximity [13] and in addition are associated with other autoimmune disorders such as Multiple Sclerosis (MS), Rheumatoid Arthritis (RA), Crohn’s Disease (CD) and Inflammatory Bowel Disease (IBD) [13, 34–37]. Additionally, we annotated the filtered variants using significant cis eQTL data across all tissues from the Genotype-Tissue Expression (GTEx) project [38]. We found that 16 filtered SNPs are annotated as the eQTL to their nearest loci while 15 filtered SNPs are annotated as the eQTL to their 3D interacting genes (Additional file 3: Table S3). These data confirmed the ability of 3DFAACTS-SNP to identify potential disease-associated regulatory region–target gene networks in a cell type-specific manner.

In addition to the annotation of the 36 T1D SNPs to 14 genes in closest linear proximity, 3DFAACTS-SNP identified 266 interacting regions and a further 47 genes that interact with the variant-containing regulatory regions via Treg Hi-C (Table 1 and Additional file 3: Table S3). We next used the 15 states regulatory model for CD4 + CD25 + CD127− Treg primary cells from the Roadmap Epigenomics Project [30] to annotate the interacting regions. Of 266 regions, 145 of them overlap with the chromHMM states that associate with transcription and gene regulation, such as transcription (4_Tx) and weak transcription states (5_TxWk), which overlaps with 71 and 124 interacting regions, respectively, and enhancers states (7_Enh), which overlaps with 88 interacting regions. We then used the Tregs chromHMM states, induced Treg super-enhancers from SEdb [39] and Tregs expressed genes from T1D patients [40] to annotate the 3D interacting genes. Of these 47 interacting genes, 38 overlapped with a Treg active chromHMM state, 14 overlapped with Treg super-enhancers and 25/47 are differentially expressed in Tregs in T1D patients [40] (Additional file 3: Table S4). Of these 25 genes 8 (CTLA4, ICOS, CCR5, BACH2, IL2RA, RASGRP1, CLEC16A and PTPN2) have been shown to be significantly associated with T1D previously [32] while our analysis has identified a further 17 new candidate genes that may be disrupted in a Treg in T1D. These data indicate that distal interacting regions contain regulatory regions and genes important for Treg function and are consistent with a model in which the variant-containing regulatory regions may contribute to T1D by disrupting the regulation of these distal interacting genes.

### The topological neighbourhood surrounding filtered T1D variants

**Fig. 2:**
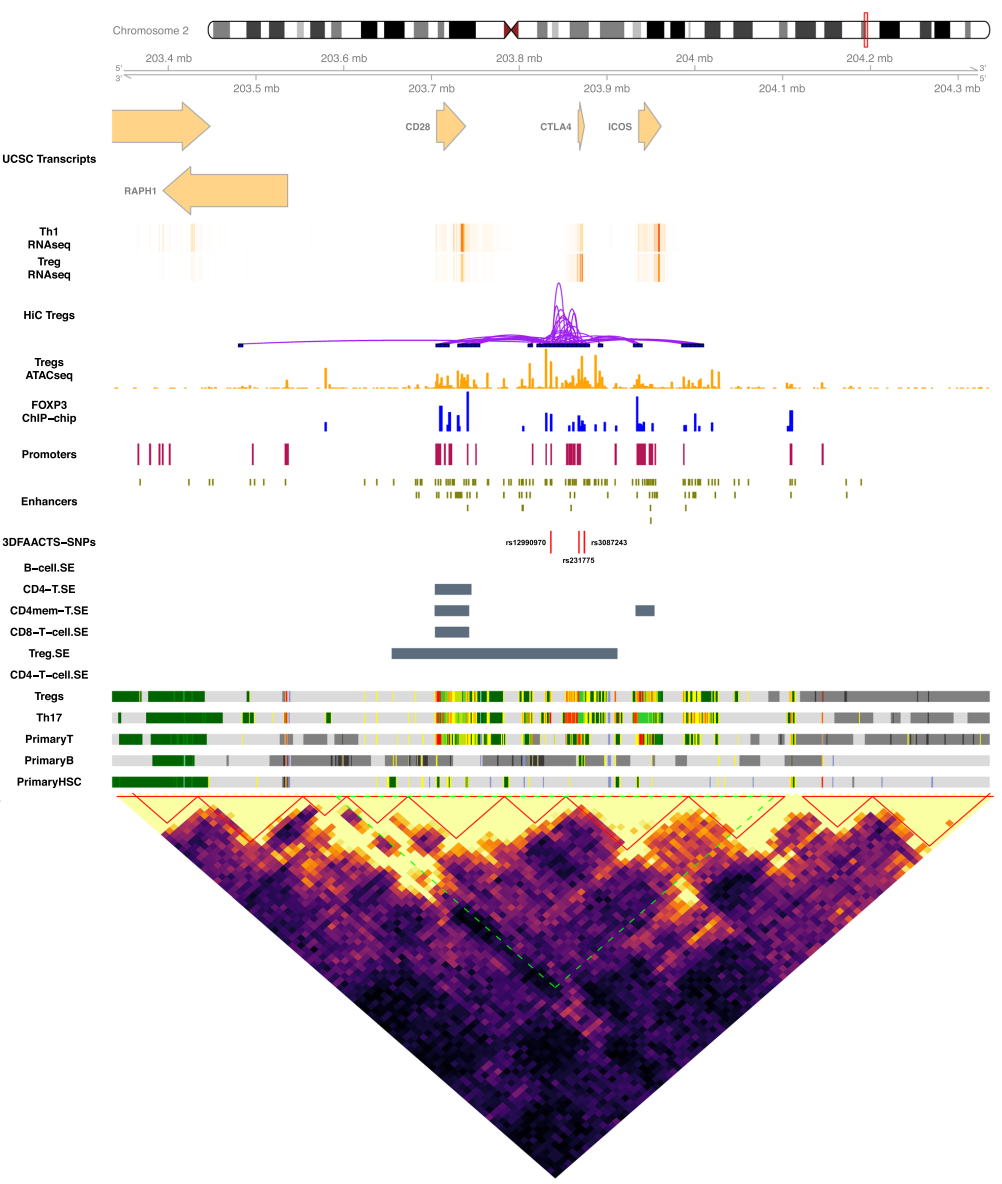
Visualisation of the CTLA4 region of filtered T1D SNPs on chr2: 203335966–204335966. Tracks displayed below the chromosome 2 ideogram display workflow datasets (3DFACCTS-SNP filtered SNPs, FOXP3-binding sites, Treg ATAC-seq peaks, statistically significant Hi-C interactions in Tregs (5 kb resolution), promoter and enhancer annotation) along with cell type-specific data including UCSC Gene Transcript information, T cell subsets (Thelper1 and Treg) expression data, super-enhancer data, 15-state ChromHMM track of T cell lineages. A heatmap showing the Tregs Hi-C interaction matrix (20 kb resolution) is located below the tracks. The plotted region of the tracks (chr22: 203335966–204335966) is indicated by the green area in the heatmap. The red triangles indicate topologically associated domains (TADs) called from the Hi-C interaction data

**Fig. 3:**
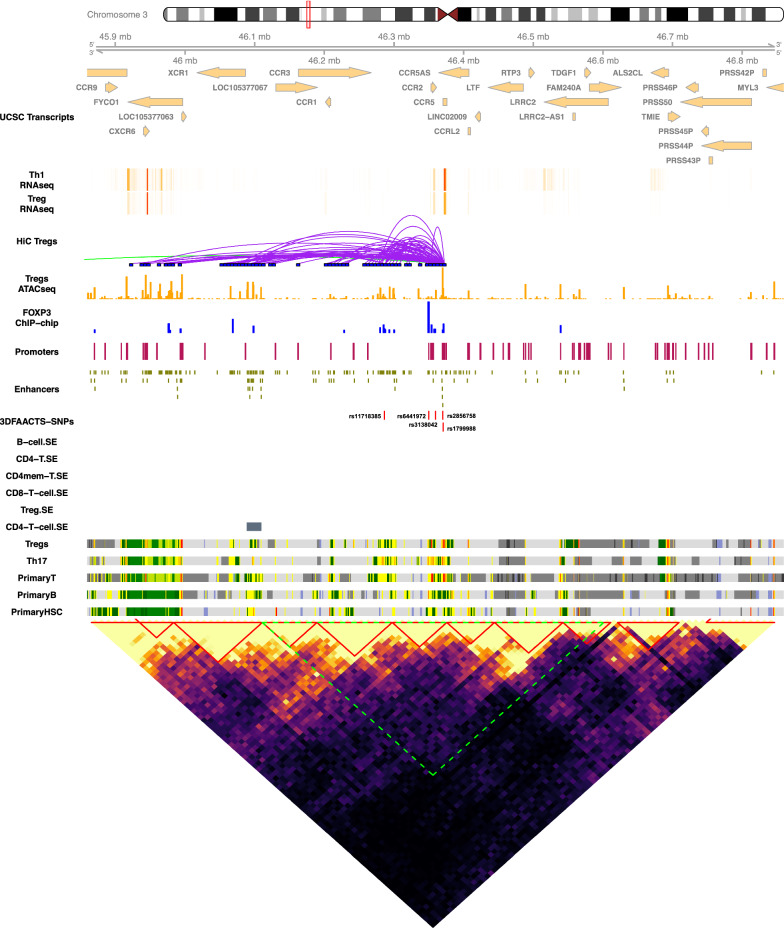
Visualisation of the CCR3/2/5 region of filtered T1D SNPs on chr3: 45859541–46859541. Tracks displayed below the chromosome 3 ideogram display workflow datasets (3DFACCTS-SNP filtered SNPs, FOXP3-binding sites, Treg ATAC-seq peaks, statistically significant Hi-C interactions of Tregs (5 kb resolution), promoter and enhancer) annotation along with cell type-specific data including UCSC Gene Transcript information, T cell subsets (Thelper1 and Treg) expression data, super-enhancer data, 15-state ChromHMM track of T cell lineages. A heatmap showing the Tregs Hi-C interaction matrix (20 kb resolution) is shown below the tracks. The plotted region of the tracks (chr3: 45859541–46859541) are indicated by the green area in the heatmap. The red triangles indicate the topologically associated domains (TADs) called from the Hi-C interaction data

We next investigated the topological neighbourhood, i.e. the presence of topologically associated or frequently interacting domains, in which regulatory regions harbouring the filtered T1D variants reside. By establishing putative boundaries of each 3D structural domain, we are then able to characterise the coordination of contacts within a locus and how they act to control gene expression. We called topologically associated domains (TADs) using Treg Hi-C interactions of 20-kb resolution (Additional file 3: Table S5) and integrated with publicly available super-enhancer, chromHMM data of T cell lineages and Treg expression data [41]. All data were overlapped across each locus and displayed in Figs. 2, 3, 5 and Additional file 1: Fig. S4–S12.

Chromatin interactions between genes and enhancer regions was detected within the variant-containing TADs, with these interactions both confirming previously identified SNP–target combinations and indicating potential new targets for investigation. For example, 3DFAACTS-SNP identified rs12990970 (chr2:203,835,966) as a potential causative T1D SNP. In Treg cells, rs12990970 is found in a flanking active TSS (TssAFlnk) state and it is located within a Treg super-enhancer (Fig. 2). This variant is located in a non-coding region between gene CTLA4 and CD28 and in past studies, and it has been associated with CTLA4 as it is an eQTL for CTLA4 expression in testis, although not in T lymphocytes or whole blood (Additional file 3: Table S3) [11, 13, 38, 42]. While Hi-C interaction signals indicate that the rs12990970-containing region interacts with the CTLA4 promoter in Treg, additional Hi-C interactions indicates that this region also form interactions with the promoter and enhancer regions connected to the costimulatory receptor CD28 gene and the inducible costimulatory ICOS gene as well (Table 1 and Fig. 2). CD28 is a family member known to play a critical role in Treg homeostasis and function [43] and ICOS is believed to play an essential part in the suppressive function of Tregs [44], suggesting CD28 and ICOS are potential novel functional targets for this variant in Treg in T1D. Finally, additional contacts between the regulatory regions harbouring variants rs231775 and rs3087243, respectively, and the RAPH1 gene were identified. In mice the adaptor protein RAPH1 has recently been shown to play an important Treg-specific role in integrin activation, Treg-suppressive function and Treg homing to the gut in a mouse model of colitis [45] suggesting changes in RAPH1 expression associated with regulatory regions harbouring variants rs231775 and rs3087243 may contribute to Treg defects in humans.

Another example of novel T1D-linked functional annotation is on chromosome 3, where Hi-C interactions indicated that the chemokine receptor genes CCR1, CCR2, CCR3, CCR5 and CCR9 (Fig. 3) are extensively linked in a set of continuous TADs, indicating that these genes may be coordinately regulated. This is supported by previous RNA Pol-II ChIA-PET work [46] that detected interactions between chemokine gene clusters during immune responses including an increase in interactions amongst the CCR1, CCR2, CCR3, CCR5 and CCR9 genes during TNF stimulation of primary human endothelial cells [46] (Additional file 1: Fig. S13). In support of this we found extensive Hi-C contacts between variant associated regulatory regions and CCR1, CCR2, CCR3 and CCR5 genes in Treg cells. Recently, CCR2, CCR3 and CCR5 have been shown to have additional chemotaxis-independent effects on Treg cells with individual studies, reporting positive roles for individual chemokine receptors on CD25, STAT5, and FOXP3 expression and Treg potency [47–49], highlighting the importance of multiple genes at this locus on Treg function. The chemokine receptor XCR1 gene also contacted by regulatory regions harbouring T1D associated variants rs11718385, rs2856758 and rs1799988 has also been implicated in Treg defects in human allergic asthma, with reduced XCR1 expression on CD4 + CD25highCD127low/ − regulatory T cell (Treg) shown to be associated with impaired regulatory function [50].

### Filtered T1D variants are enriched at lineage-specific T cell super-enhancers

**Fig. 4:**
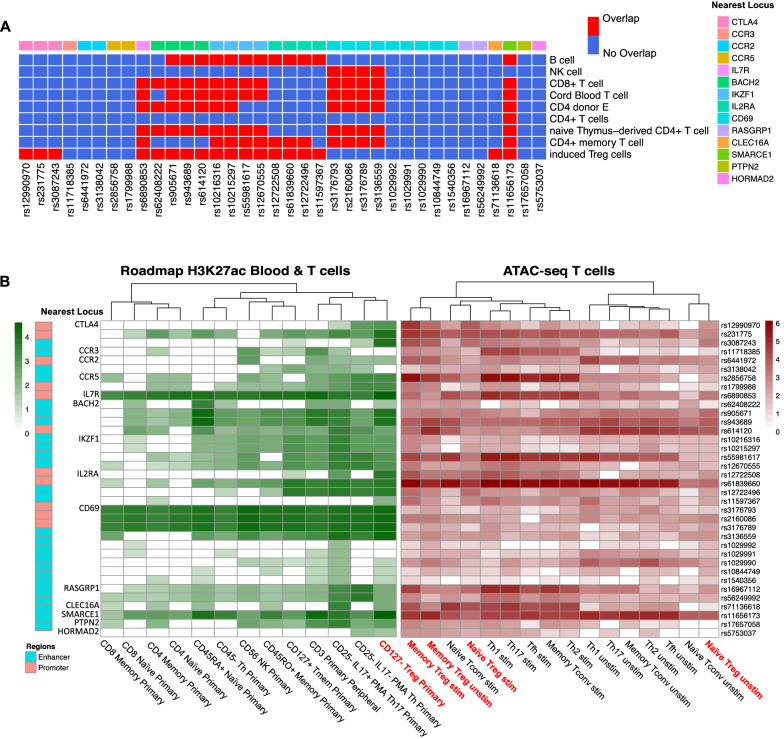
Integrating T1D 3DFAACTS SNPs with different data of T cell lineages. **A** Heatmap showing overlapping status between T1D 3DFAACTS SNPs and super-enhancers of different T cell lineage from SEdb [39], where red indicates variants overlapping with SEs and blue indicates not overlapping. **B** Enrichment of filtered T1D variants found within H3K27ac peaks from Epigenomics Roadmap and ATAC-seq peaks from multiple T cell lineages [30]. Column names in red indicates Tregs specific datasets

SEs usually consist of a cluster of closely spaced enhancers that are defined by their exceptionally high level of transcription co-factor binding and enhancer-associated histone modifications (i.e. H3K27ac) compared to all other active enhancers within a specific cell type [51]. SEs are also linked to the control of important processes such as cell lineage commitment, development and function [52]. Analysing T cell SE information annotated in the Super-Enhancer Database [39] (SEdb), 8 out of the 14 variant-containing loci were found to contain filtered T1D variants located in SEs formed in various T cell lineages including Treg cells consistent with the enrichment of autoimmune-disease associated variants within T cell super enhancers reported previously [52] (Fig. 4A). The loci containing the CTLA4 and CLEC16A genes were the only loci that overlapped with Treg-specific SEs. The existence of a Treg SE is consistent with the different regulation of CTLA4 in Treg cells compared with other T cell lineages [53] and a recent report linking T1D risk variants to altered CLEC16A expression in Treg [40]. Five other SNPs are located within SEs in multiple T cell types including induced Treg (iTreg) suggesting the gene controlled by these SE play a broad role in T cell function. While no Treg SEs are detectable at the CD69 locus the T1D associated variants in this region overlapped with SEs formed in other T subsets. No T cell-associated SEs are found in the loci containing the CCR1/2/3/5, PTPN2, RASGRP1 and HORMAD2 genes (Fig. 4A).

We then investigated the level of active enhancer marks (normalised H3K27ac-binding) and chromatin accessibility (normalised ATAC-seq peak coverage) overlapping each variant from Table 1 (Fig. 4B). A range of tissue restriction patterns of chromatin states were observed using the NIH Epigenomics Roadmap data with enhancers displaying in general a more cell type-restricted pattern of H3K27ac signal compared to promoters. No variant was found to be located in a regulatory region that was exclusively active in Treg cells although rs12990970, rs231775 (CTLA4), rs11597367, rs12722508 (IL2RA) and rs5753037 (HORMAD2) are associated with a restricted H3K27ac pattern that included Treg. The absence of Treg-specific enhancers is consistent with FOXP3 binding data where FOXP3 binds many enhancer regions active in other T cell lineages to modify their activity in Treg cells [54]. Evidence suggests FOXP3 cooperates with other T helper-lineage specifying transcription factors to diversify Treg cells into subsets that mirror the different Th-lineages [55–57]. Most regions associated with the variants show an increase in chromatin accessibility upon stimulation in Treg and T helper subsets consistent with increased enhancer activity upon T cell activation, however, in a few instances variants are in regions that decrease in accessibility in stimulated Treg and T helper subsets compared with their matched unstimulated counterpart. Notably these include the variants rs905671, rs943689 and rs614120 associated with BACH2. This is consistent with the reduction in BACH2 expression in CD4 T cells as they mature, and alteration to this repression is linked to proinflammatory effector function [58]. Together these data are consistent with a model in which causal variants alter the output of enhancers that respond to environmental cues [59].

### Filtered variants disrupt transcription factor binding sites (TFBS) including a FOXP3-like binding site

Fundamental to understanding the function of specific disease-associated variants is the identification of the potential impact of these non-coding variants on transcription factor binding. Analysis of ATAC-seq datasets with HINT-ATAC [60], identified over 5 million active TF footprints in chromatin accessibility profiles from stimulated and resting Treg populations (Additional file 4). By imposing the additional FOXP3 binding annotation to the footprint dataset, we identified 7 T1D-associated variants that have the potential to alter the binding of 9 TFs, suggesting the molecular mechanisms by which these variants could impact Treg function (Additional file 3: Table S6). Of these 7 SNPs, one SNP rs3176789 is located in an active TSS chromHMM state region, while the others are located either in enhancers or flanking active TSS that are associated with active enhancers, suggesting these variants might interrupt the binding of TFs to affect enhancer functions, with the potential for a network effect on multiple genes.

We then used GWAS4D [61], which computes log-odds of probabilities of the reference and alternative alleles of a variant for each selected TF motif to calculate binding affinity, to predict the regulatory effect of each variant (Additional file 3: Table S7). Several of the variants are predicted to alter the binding of transcription factors with known roles in Treg and other T cell lineages including nuclear activator of T cells (NFATC2 and NFATC3, rs1029991) [62], interferon regulatory transcription factor (IRF, rs3176789) [63], myocyte enhancer factor 2 (MEF2, rs6441972 and rs3176789) and FOX (Forkhead box, rs614120) family members. In addition, variant (rs1029991) has the potential to alter the binding of YY1, recently identified as an essential looping factor involved in promoter–enhancer interactions [64]. Other variants (rs1136618 and rs3176789) potentially alter the binding of the zinc finger protein ZNF384. Although expressed in T cells, the importance of ZNF384 in T cell biology has not yet been explored.

**Fig. 5:**
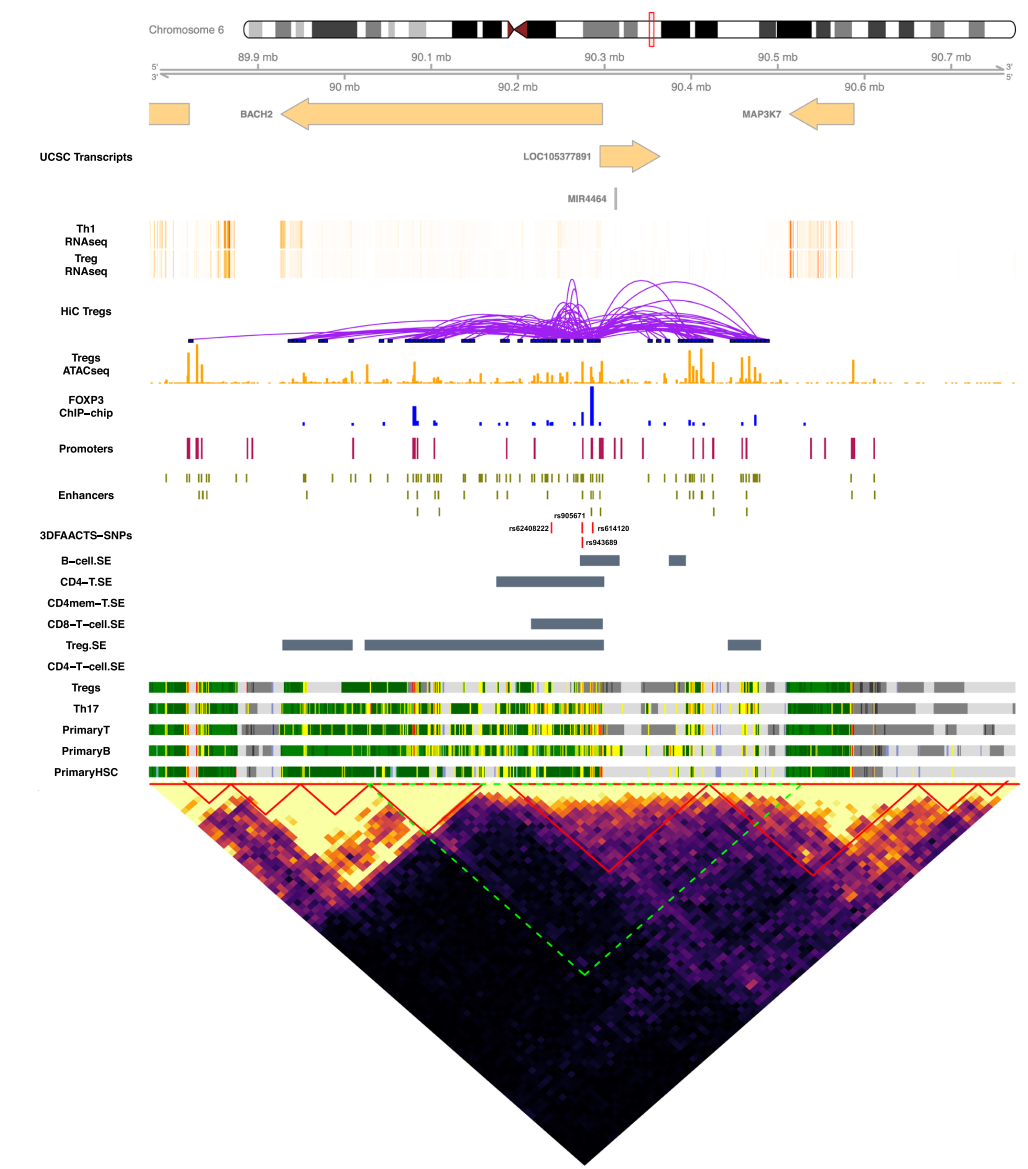
A visualisation of the BACH2 region of filtered T1D SNPs on chr6: 89774131–90774131. Tracks displayed below the chromosome 6 ideogram display workflow datasets [3DFAACTS-SNP filtered SNPs, FOXP3-binding sites, Treg ATAC-seq peaks, statistically significant Hi-C interactions in Tregs (5 kb resolution), promoters and enhancers along with cell type-specific data including UCSC Gene Transcript information, T cell subsets (Thelper1 and Treg) expression data, super-enhancer data, 15-state ChromHMM track of T cell lineages. A heatmap showing the Tregs Hi-C interaction matrix (20 kb resolution) is shown below the tracks. The plotted region of the tracks (chr6: 89774131–90774131) are indicated by the green area in the heatmap. The red triangles indicate the topologically associated domains (TADs) called from the Hi-C data

Of note, rs614120 is predicted to decrease the binding affinity of FOXA2 in this enhancer region (Additional file 3: Table S8). As FOXA2 is not expressed in the immune compartment, this SNP may interfere with the binding of another member of the forkhead class of DNA-binding proteins, e.g. FOXP3, which is localised to this region based on our FOXP3 ChIP (Fig. 5). This suggests that a model in which rs614120 impacts the expression level of BACH2 and/or AFG1L is by altered binding of a FOX protein to this enhancer.

Filtered variant rs1029991 is predicted to alter the binding of NFAT family members and/or YY1 to the enhancer region, but we have been unable to link the associated regulatory region which harbours this variant to any gene. Filtered variant rs3176789 is predicted to alter IRF and/or MEF2 binding, linking these transcription factors to the regulation of the CLEC family member CLEC2D and two additional genes LOC374443 (C-Type Lectin Domain Family 2 Member D Pseudogene) and LOC105369728 a putative lncRNA class gene. Examination of gene expression data from Treg indicate that only CLEC2D is expressed in a Treg (Additional file 3: Tables S3 and S4). The CD69 and CLEC2D genes have previously been associated with T1D by GWAS, however we could not detect any significant interaction of regulatory regions harbouring 3DFAACTS-SNP filtered SNPS and the CD69 gene. Filtered variant rs6441972 is also predicted to influence the binding of MEF2 to a regulatory region in proximity to the promoter of CCR2. Consistent with this variant disrupting CCR2 expression, CCR2 is a target gene for eQTL rs6441972, indicating that rs6441972 may result in altered CCR2 expression in a Treg in T1D by interfering with MEF2 binding.

### Filtered Treg variants identified in other autoimmune diseases

The primary rationale of our filtering workflow is that autoimmune diseases like T1D are mediated by altered Treg functions. Hence, using GWAS data for other autoimmune diseases, we aimed to discover variants which potentially act by disrupting 3D gene regulation in Tregs. Like filtering fine-mapped T1D-associated SNPs, here we used the 3DFAACTS-SNP filtering workflow to process variants identified by Immunochip fine-mapping experiments and meta-analysis from three studies for a broad range of autoimmune and inflammatory diseases. SNPs associated with 10 autoimmune diseases were identified, representing 221 fine-mapped SNPs associated with multiple sclerosis (MS) [65]; 69 SNPs identified by the meta-analysis of celiac disease (CeD), rheumatoid arthritis (RA), systemic sclerosis (SSc), and T1D [66] (which we refer to the 4AI dataset); and 244 SNPs identified by the meta-analysis of GWAS datasets for ankylosing spondylitis (AS), Crohn’s disease (CD), psoriasis (PS), primary sclerosing cholangitis (PSC) and ulcerative colitis (UC) [67] (which we refer as 5ID dataset). Applying the 3DFAACTS-SNP pipeline we identified 9, 3 and 6 filtered variants from the MS, 4AI and 5ID datasets, respectively (Additional file 3: Table S9). We identified putative target genes for these disease associated variants by Hi-C interactions resulting in 34, 8 and 23 genes (60 unique genes) linked to MS, 4AI and 5ID, respectively. Of these 34/60 are differentially expressed in Tregs isolated from T1D patients compared to healthy controls highlighting the potential of 3DFAACTs-SNP to identify candidate SNP–target gene interactions in disease (Additional file 3: Table S9). Many of these genes have either known roles in Treg differentiation, stability and function (CD48, IL6ST, EZR, ELMO1, IL18R1) [68–72], or altered expression in human Treg in autoimmune disease (SLAMF1, ANKRD55, TAGAP) [73–75].

Of the variants identified by 3DFAACTS-SNP, one variant (rs60600003) located at a locus on chromosome 7 was found to be associated with several diseases, including MS [65], celiac and systemic sclerosis [66], suggesting its interacting gene, ELMO1, may contribute to a common Treg defect in these diseases (Additional file 3: Table S9). When compared with the 36 variants identified from our T1D dataset analysis, two variants, rs61839660 on chromosome 10 and rs3087243 on chromosome 2 were also prioritised by 3DFAACTS-SNP analysis of the 5ID and 4AI datasets, respectively, implicating their interacting genes IL2RA, RBM17, PFKFB3 and LINC02649 (rs61839660), RAPH1 (rs3087243) are functionally implicated in the development of these diseases. While different variants were identified in the analysis of the various disease datasets, the regulatory elements in which these variants reside can be linked by Hi-C data to common candidate target gene such as PFKFB3 (rs12722496 from T1D and rs947474 from 4AI) and RAPH1 (rs231775 from T1D and rs3087243 from 4AI). This is consistent with the view that common mechanistic pathways underlie some autoimmune diseases, although the specific risk allele within a locus can be disease-specific [76].

### Identifying new variants that are candidates for impacting autoimmune disease

Most variants identified by GWAS have small effect sizes that together only represent a fraction of the heritability predicted by phenotype correlations between relatives [77]. To account for this missing heritability, various models have been proposed including a highly polygenic architecture with small effect sizes of the causal variants [78, 79], rare variants with large effect size [80, 81] and epistatic mechanisms including gene–gene and gene–environment interactions [82, 83]. As a consequence many causal variants with small effect sizes are unlikely to reach genome wide significance in current GWAS, whereas rare variants are often under-represented on SNP arrays [84]. Lastly the preponderance of studies utilise populations of European descent which can result in a bias for SNPs with a higher minor allele frequencies in Europeans compared to other populations, potentially limiting the relevance of these SNPs to the associated traits in non-Europeans [85]. As an alternative approach, to identify novel putative autoimmune disease-associated SNPs independently of association studies, we sampled 5,888,594 common variants (MAF > 0.1) from the Genome Aggregation Database (gnomAD) (version 3.0) [86] as inputs to our filtering workflow, identifying a total of 9376 gnomAD-3DFAACT SNPs (Additional file 3: Table S10).

**Fig. 6:**
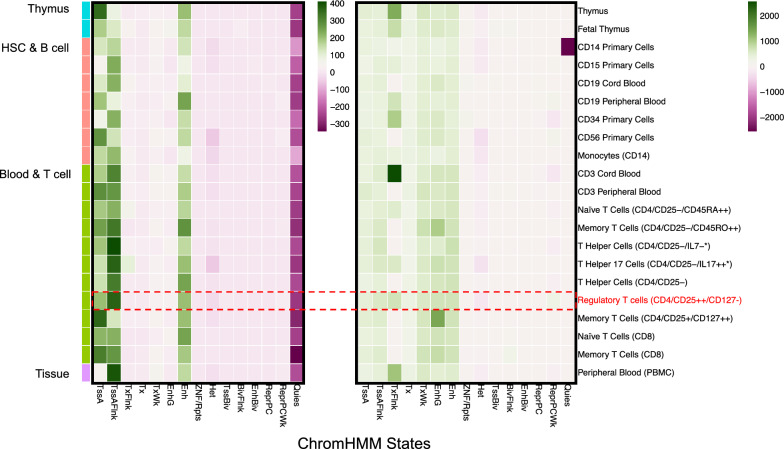
Enrichment of gnomAD 3DFAACTS SNPs (left panel) and their interacting regions (right panel) found within NIH Epigenomics Roadmap samples. Enrichment test of filtered gnomAD SNPs against chromHMM states from 129 tissues and cell types from Epigenomics Roadmap using GIGGLE [87]. Green coloured regions indicate positive enrichment of variants within cell types and chromHMM states, while purple-coloured regions indicate negative enrichment. Here we subset to enrichment in three tissue groups, including thymus, HSC and B cell and blood and T cell, enrichment results of all samples can be found in Additional file 1: Fig. S14 and S15

In order to characterise the SNPs, we used *GIGGLE* [87] to compare the regions in which filtered SNPs reside against 15 predicted chromHMM genomic states across 127 cell types and tissues from Epigenomic Roadmap [30] (Fig. 6 left and Additional file 1: Fig. S14), calculating positive and negative enrichment scores according to overlapping sets. Interestingly, there was strong positive enrichment signal in active TSS (*TssA*), flanking active TSS (*TssAFlnk*) and enhancers (*Enh*) states in thymus, HSC, B- and T- cell groups, while only enrichment of TssA was observed across all cell types, and enrichment of TssAFlnk and Enh are seen in only immune-related cell types. This suggest that the enhancer regions related to our identified SNPs are highly specific to immune cells while the promoter regions and by extension their target genes are broadly expressed (Additional file 1: Fig. S14). Additionally, negative enrichment of the quiescent (*Quies*) states was observed in all cell types whereas heterochromatin (Het) exhibited a negative enrichment in some specific cell types (foreskin fibroblast primary cells) and cell lines (IMR90, HVEC and HMEC).

Treg Hi-C data were used to explore the FOXP3-associated regulatory networks that include these SNPs in a Treg. For the regions identified to interact with the 9,376 variants located in FOXP3 binding regions by Hi-C, we observed positive enrichments of regulatory states such as *TssA*, *TssAFlnk*, *Tx*, *Txwk*, *EnhG* and *Enh* in blood, HSC, B and T cells, supporting a regulatory role for these interacting regions (Fig. 6 right and Additional file 1: Fig. S15). 4,968 genes are found to interact with the identified filtered gnomAD-3DFAACTS SNPs via the Treg Hi-C interactions (Additional file 3, Table S10). Gene set enrichment analysis of these genes was performed using the Hallmark genes sets and gene ontology (GO) terms from the Molecular Signatures Database (MSigDB) [88]. Significantly enriched (adjusted *P*-value < 0.05) gene sets that are highly biological relevant were found, including T cell activation, regulation and differentiation GO terms and autoimmune/Tregs-related gene sets, including TNFα via NF-κB, IL6/JAK/STAT3, and IL2/STAT5 signalling pathways (Additional file 1: Fig. S16). Together, these data indicate that using the 3DFAACTS SNP pipeline in combination with the gnomAD database has the potential to prioritise potential functional variants in a specific cell type and identify their interacting genes with potential molecular mechanisms of action.

## Discussion

GWAS and fine-mapping studies have identified over 50 candidate regions for T1D progression [11, 13], however a broad understanding of the underlying disease mechanism has been difficult to elucidate without relevant functional information derived from cell-specific material. With the availability of whole genome annotation, we see that the majority of genetic risk lies in non-coding regions of the genome and is enriched in regulatory regions including promoters and enhancers. Traditionally, to understand how these variants may function they have been assigned to the nearest gene or genes within a defined linear distance. However, this approach ignores the role of three-dimensional connectivity by which enhancers and repressors function to regulate transcription [89–91].

Recent approaches use statistical co-localisation tests to link potential causal SNPs and quantitative trait loci (QTLs) to identify the genes regulated by GWAS loci [92]. These methods require many samples in the correct cell type or physiological context and to date work best for local/*cis* QTLs, generally less than 1 Mb in linear distance [89]. An alternative approach used in this study and others [93, 94] is to make use of chromosome conformation capture data to directly connect disease-associated regulatory regions to their target genes. As growing cellular and genomics evidence indicate that dysregulation of the Treg compartment contributes to autoimmune disease [25, 95, 96], we generated a cell type-specific 3D interaction profile in human regulatory T cells to establish an in silico*,* candidate loci reduction method to identify T1D-candidate regions that function in a Treg and the genes they affect. Open chromatin regions identified by ATAC-seq and regulatory regions identified by epigenetic marks such as histone H3K27ac can number in the tens of thousands in a specific cell type [40, 97], we therefore initially focused on regulatory regions bound by the Treg-specific transcription factor FOXP3 given the essential role of FOXP3 in the Treg functional phenotype we hypothesised that candidate variants that are found within open, FOXP3-bound regions are likely to alter immunological tolerance. In addition, as different autoimmune diseases share genetic risk regions [13], we speculated that by identifying specific genetic variants that may contribute to T1D through the dysregulation of regulatory T cell functional fitness, this could be via mechanisms consistent across many autoimmune diseases [1, 98, 99].

The design and implementation of the 3DFAACTS-SNPs workflow champions a new data-centric view of functional genomics analysis, with the development of cell type-specific epigenomic and 3D datasets enabling researchers to narrow down on molecular changes at a fine-scale resolution. However, the results of this study suggests that cell type-specific viewpoints can be broadened to a much more lineage (T cell) or immune (e.g. innate or adaptive) system-specific level. While we focused on Treg cells and expected to identify Treg-specific enhancer-controlled targets, based on the criteria of inclusion of FOXP3 binding data, no functional variant was uniquely accessible in only Tregs, nor were they specifically enriched with Treg-exclusive H3K27ac modified regions (Fig. 4B). This likely reflects the propensity of FOXP3 to bind to enhancers active in multiple CD4 + T cell lineages [54] (Fig. 4) to modify their output in a Treg-specific manner and therefore we cannot currently discern whether these filtered variants act predominantly in Tregs or on other CD4 + T cell subsets. The incorporation of context- and CD4 + T cell subset-specific gene expression [100] and epigenomic [94, 101] data into the 3DFAACTS-SNPs workflow may help resolve this. Although we have focused here on using FOXP3-binding as a filtering criteria, it is known that other FOXP3-independent pathways are important for Treg function and the 3DFAACTS-SNPs workflow could be modified to incorporate other TFs or other epigenetic profiles such as CpG-demethylated regions [102] to further explore the relationship between disease-associated variants and these pathways.

In total using the 3DFAACTS-SNPs workflow we identified 47 novel candidate genes connected to variants in 12 T1D risk loci that could plausibly function in a Treg whereas we could not define plausible candidate Treg-specific activity at the other T1D risk regions that met all our filtering criteria. This may indicate that these other risk-regions are active in immune cell types other than a Treg or they impact genes and regulatory elements within a Treg that are not dependent upon FOXP3. As an example of how the 3DFAACTS-SNPs workflow can lead to testable insights into the molecular mechanisms of non-coding variants, the SNP rs614120 was found to be located in a FANTOM5 annotated T cell-specific enhancer region in the first intron of the BACH2 gene, and is predicted to disrupt the binding of Forkhead Transcription factor family member FOXA2 (Fig. 5 and Additional file 3: Table S6). However, FOXA2 is not expressed in T cells, indicating that rs614120 might disrupt the binding of other Forkhead family members which bind to very similar DNA sequences, such as FOXP3, which is known to bind in this region (Fig. 5). The 3DFAACTS-SNPs workflow further indicates that this enhancer region containing rs614120 interacts with the promoter of BACH2, forming a distal promoter–enhancer interactions, suggesting that rs614120 may disrupt FOXP3 binding to the enhancer leading to the dysregulation of BACH2 expression. It has been recently shown that Bach2 plays roles in the regulation of T cell receptor signalling in Tregs, including averting premature differentiation and assisting peripherally induced Treg development [103]. Therefore, we suggested that this single variant may regulate BACH2 expression and ultimately may affect the progression of T1D, and this requires further experiments to verify. This can further aid the development of novel therapeutic approaches to restore function in Treg of patients with this genotype. This finding also suggests that variants can contribute to the causal mechanisms of disease by altering the efficacy/stability of TF binding in important regions such as enhancers or SEs.

The power of 3DFAACTS-SNPs is its ability to incorporate chromosome organisation in 3D and identify long-range interactions involving variant-containing regulatory regions leading to the identification of target genes that have not previously been associated with these diseases associated risk regions. This is illustrated by the finding that the majority (39/46) of the genes that interact with the T1D variants are not the closest gene in linear proximity and of these interacting genes some have not been previously associated with any autoimmune disease.

The idea that high-order nuclear organisation coordinates transcription in times of immune challenge or tolerance was recently shown in a study demonstrating that 3D chromatin looping topology is important for a subset of long non-coding RNAs (lncRNAs), termed immune gene-priming lncRNAs (IPLs), to be correctly positioned at the promoters of innate genes [46]. This positioning of the IPLs then allows for the recruitment of the WDR5–mixed lineage leukaemia protein 1 (MLL1) complex to these promoters to facilitate their H3K4me3 epigenetic priming [46]. An example of long-range enhancer gene interactions in conveying autoimmune-disease risk in Treg cells has also recently been published [104]. In this work a distal enhancer at the 11q13.5 locus associated with multiple autoimmune-disease risk, including T1D was found to participate in long-range interactions with the LRRC32 gene exclusively in Treg. Deletion of this enhancer in mice resulted in the specific loss of Lrcc32 expression in Treg cells and the inability of Treg to control gut-inflammation in an adoptive transfer colitis model. Furthermore CRISPR-activation experiments in human Tregs identified a regulatory element located in proximity to a risk variant rs11236797 that is capable of influencing LRRC32 expression. This data together highlights the mechanistic basis of how non-coding variants may function to interfere with Treg activity in disease. This interaction was present in our Hi-C dataset, but it was filtered out as the enhancer is not bound by FOXP3, showing the cell type-specific filtering power, as well as conserved connectivity in T cells. Coordinated genome topology has also been shown in immune cell lineage commitment, both at a locus [105, 106] and compartment level [107], consistent with the concept of immune transcriptional “factories” where genes congregate in regions of the nucleus to undergo coordinated transcriptional activation [108].

Although a shared genetic aetiology between T1D and other immune-mediated diseases has been proposed, we did not find a large overlap between the variants or interacting genes identified by 3DFAACTS SNP in T1D and other autoimmune disease datasets. The reason for this is not clear, but may be a result of the relatively low number of input SNPs for the other autoimmune diseases. Irrespective of this, two candidate causal SNPs and genes including rs3087243 (RAPH1) and rs61839660 (IL2RA, RBM17, PFKFB3, LINC02649) were found to be common between T1D and other autoimmune diseases. Several of these genes such as IL2RA and PFKFB3 have previously been implicated in the development of autoimmune diseases or play a role in critical T cell pathways, suggesting these genes are likely targets that explain the molecular function of the risk variants. PFKFB3 is involved in both the synthesis and degradation of fructose-2,6-bisphosphate, a regulatory molecule that controls glycolysis in eukaryotes. Regulation of glycolysis has increasingly been implicated in shaping immune responses [109] and PFKFB3 has been associated with multiple autoimmune diseases [110]. Importantly, reduced PFKFB3 enzyme activity leading to redox imbalance and apoptosis has been reported in CD4 + T from RA patients [111] directly linking the PFKFB3 gene to the disease. This provides molecular insight into environmental pressure on immune cell function driving loss of tolerance.

A highly polygenic architecture with small effect sizes of many causal variants [78, 79] has been proposed to account for missing heritability associated with phenotypic traits. Most of these small effect size variants have yet to be identified. Here we have begun to investigate whether common genetic variation found within populations could contribute to autoimmune diseases by altering gene-expression by altering enhancer and promoter output. In this study we illustrate this potential by accessing large population-scale variant resources in the gnomAD database, identifying 9376 filtered common variants that have the potential to impact Treg function. Based on the search of discovered associations of autoimmune diseases (EFO_0005140) from the GWAS Catalog [112], over half of the variants surveyed here have not been used in large-scale autoimmune disease GWAS [11, 67, 113–119], precluding their assessment for potential disease risk in sampled disease/control populations. While filtered variants identified here are biased towards the inclusion of FOXP3-binding within the workflow, their potential immune response impact is highlighted by the finding that their interacting regions are positively enriched for transcription and enhancer-associated chromatin states (Fig. 6, Additional file 1: Fig. S14 and S15). This accessibility of regulatory variants among a population could potentially explain additional variation in effector responses in T cell activation [120], relevant not only to autoimmune disease, but also to broader immune responses for example to SARS-CoV-2.

In conclusion, while we initially restricted the application of 3DFAACTS-SNP to Treg-centric genome-wide interaction frequency profiles to give functional annotation in T1D data, we have demonstrated that valid interacting pairs from Hi-C dataset can be functionally mapped with high confidence from multiple disease datasets as well as whole genome variant datasets, which presents a valuable resource in establishing cell type-specific interactomes. Coupled with cell type-specific genomic data available from public repositories, such as the NIH Roadmap [30], Blueprint [121] and ENCODE [122] projects, this workflow provides a useful mechanism to identify potential mechanisms by which non-coding variants regulate disease causing genes, and identifies new targets for therapeutic modulation to treat or prevent disease.

## Conclusion

Based on Treg ATAC-seq, Hi-C data, promoter and enhancer annotation and FOXP3 binding site annotation, we have developed a variant filtering workflow named 3DFAACTS-SNP to identify potential causative SNPs and their 3D interacting genes for T1D from GWAS fine-mapped variants. Our workflow can easily be used with variants associated with other autoimmune diseases or even large population-scale variants.

## Methods

### Cell preparation

Peripheral blood mononuclear cells (PBMCs) were isolated from whole blood obtained from healthy human donors with informed consent at the Women’s and Children’s Hospital, Adelaide (ethics approval and consent see “Declarations” section). Cells were labelled with the following fluorochrome conjugated anti-human monoclonal antibodies: anti-CD4 (BD Biosciences, BUV395 Mouse Anti-Human), anti-CD25 (BD Biosciences, BV421), anti-CD127 (BD Biosciences, PE-CF594) and viability dye (BD Biosciences, BD Horizon Fixable Viability Stain 700) for FACS analysis by surface expression staining. Regulatory T (Treg) cells were sorted as CD4 + CD25hi CD127dim population (> 90% purity). Following cell sorting Treg cells were plated at 100,000 cells per well in a 96-well U-bottom plate and maintained in complete X-VIVO 15 culture media (X-VIVO 15 Serum-free media supplemented with 2 mM HEPES pH 7.8, 2 mM l-glutamine and 5% heat inactivated human serum) in 400 U/mL rIL-2 for 2 h at 37 °C in a humidified 5% CO_2_ incubator prior to cell preparation for ATAC-seq experiment.

### ATAC-seq library preparation and high-throughput sequencing

Treg cells were rested for 2-h post-sort and then were either left untreated or stimulated with beads conjugated with anti-CD3 and anti-CD28 antibodies (Dynabeads Human T-Expander CD3/CD28, Gibco no. 11141D, Life Technologies) in complete X-VIVO 15 culture in 400 U/mL rIL-2 at a cell/bead ratio of 1:1 for 48 h. After 48 h Dynabeads were removed from culture medium by magnetic separation. Omni ATAC-seq was then performed as described previously [154] with minor modifications. Briefly, cells with 5–15% dead cells were pre-treated with 200 U/µL DNase (Worthington) for 30 min at 37 °C prior to ATAC-seq experiments. Treg cells (50,000) were lysed in 50 µL of cold resuspension buffer (RSB: 10 mM Tris–HCl pH 7.4, 10 mM NaCl, and 3 mM MgCl_2_) containing 0.1% NP40, 0.1% Tween-20, and 0.01% digitonin on ice for 3 min. The reaction was then washed with 1 mL of ATAC-seq RSB containing 0.1% Tween-20 by centrifugation at 500 xg for 10 min at 4 °C and the nuclei were resuspended in 50 µL of transposition mix (30 µL 2 × TD buffer, 3.0 µL Tn5 transposase, 16.5 µL PBS, 0.5 µL 1% digitonin and 0.5 µL 10% Tween-20) (Illumina Inc). The transposition reaction was incubated at 37 °C for 45 min in a thermomixer with 1000 rpm mixing. The reaction was purified using a Zymo DNA Clean and Concentrator-5 (D4014) kit. All libraries were amplified for a total of 9 PCR cycles and size selection was carried out to enrich for a fragment size window of 200–900 bp prior to sequencing. Libraries were quantified by PCR using a KAPA Library Quantification Kit for NGS (KAPA Biosystems, Roche Sequencing). Barcoded libraries were pooled and sequenced on a paired-end 75-cycle Illumina NextSeq 550 High-Output platform (Illumina) to an average read depth of 37.1 million read pairs (± 4 million) per sample.

### Treg sample preparation, Hi-C library production and high-throughput sequencing

Cord blood was obtained with informed consent at the Women’s and the Children’s Hospital, Adelaide (HREC1596; WCHN Research Ethics Committee). Mononuclear cells were isolated from cord blood postpartum as previously described [123]. Briefly, cord blood CD4^+^CD25^+^(Treg) were isolated from purified mononuclear cells using a Regulatory CD4^+^CD25^+^T Cell Kit (Dynabeads; Invitrogen, Carlsbad, CA). Ex vivo expansion of isolated T cell populations (1 × 10^6^ cells per well in a 24-well plate) were performed in X-Vivo 15 media supplemented with 5% human AB serum (Lonza, Walkersville, MD), 20 mM HEPES (pH 7.4), 2 mM l-glutamine, and 500 U/mL recombinant human IL-2 (R&D Systems, Minneapolis, MN) in the presence of CD3/CD28 T cell expander beads (Dynabeads; Invitrogen; catalogue no. 111-41D) at a bead-to-cell ratio of 3:1. Cell harvesting, formaldehyde cross-linking (2%) and nuclei isolation was per [156,157]. Treg cell nuclei were frozen in aliquots of 1 × 10^7^. The in situ Hi-C procedure was carried out as per Rao et al., (2014) [124] with the following modifications MboI digestion was carried out in CutSmart^®^ Buffer (NEB) and biotin-14-dCTP (Invitrogen; catalogue no. 19518018) replaced biotin-14-dATP in the reaction to end-fill MboI overhangs. To generate DNA suitable for library construction ligated DNA in TE buffer (10 mM Tris–HCl, pH8.0 and 0.1 mM EDTA, pH 8.0) was sheared to an average size of 300–500 bp using a Covaris S220 (Covaris, Woburn, MA) instrument with the following parameters; 130ul in a microTube AFA fibre, 140 peak incidence power, 10% Duty cycle 10%, 200 cycles per burst for 55 s. Sheared fragment ends were made suitable for adapter ligation with a NEBNext^®^ Ultra II End Repair/dA-Tailing Module (NEB #E7546). For adapter ligation the End Prep reaction was split into two and appropriately diluted NEBNext Adaptor ligated to fragment ends using the NEBNext Ultra II Ligation module. Library size distribution was determined using an Experion DNA 1K kit and library concentration estimated by real-time qPCR using a Kapa universal Library quantitation kit (Roche Sequencing Solutions; 07960140001). Hi-C libraries were sequenced on an Illumina NovaSeq™ 6000 Sequencing System (2 × 150 bp).

### ATAC-seq data analysis

The sequencing data quality was determined using *FastQC* (ver. 0.11.7) [159] followed by trimming of Nextera adapters using *cutadapt* (ver. 1.14) [125]. Trimmed reads were aligned to the human hg19 (hs37d5) reference genome using *Bowtie2* (ver. 2.2.9) [126] with ‘-X 2000’ setting. For each sample quality trimming was performed with option ‘-q 10’ with unmapped and non-primary mapped reads filtered with option ‘-F 2828’ using *Samtools* (ver. 1.3.1) [127]. PCR duplicates were then removed from Uniquely mapped paired reads using *Picard* (ver. 2.2.4). Mitochondrial reads, reads mapping to ENCODE hg19 blacklisted regions and mitochondrial blacklisted regions were filtered out using *BEDTools* (ver. 2.25.0). The TSS enrichment score for each replicate was determined using ATACseqQC [128]. For peak calling the read start sites were adjusted to represent the centre of Tn5 transposase binding event. Peaks were called from ATAC-seq data using *MACS2* (ver. 2.1.2) [129] with parameters ‘—atac-seq —paired-end —organism = hg19 —*P* 0.05’ [130, 131] and HINT-ATAC [60] was used to call footprints from the ATAC-seq peaks with parameters ‘—atac-seq —paired-end.

The peak summits from resting and stimulated Treg were concatenated and sorted by chromosome and then by position. The sorted peak summits were then handled using an in-house Python script *ATACseqCollapsing.py*, which adapted a peak processing approach described by Corces et al. [132] to generate a list of non-redundant peaks. Briefly, through an iterative procedure, the peak summits are extended by 249 bp upstream and 250 bp downstream to a final width of 500 bp. Any adjacent peak that overlaps with the most significant peak (significance value defined by *MACS2*) within the interval is removed. This process iterates to the next peak interval resulting in a list of non-redundant significant peaks. Finally, to be consistent with other annotations in the 3DFAACTS-SNP workflow, the ATAC peaks were lifted over to the hg38 genome.

### Hi-C data analysis

The raw sequencing read files were first aligned to the human hg38 genome using BWA-mem with setting “*-SP5M*”. The mapped read pairs with mapping quality (MAPQ) over 30 were selected to process with pairtools [133] to identify Hi-C interactions. The interactions are then mapped to genomic bins of 5 kb and 20 kb resolution using cooler [134]. The contacts of 5 kb bins were further processed to identified intra-chromosomal statistically significant (BH adjusted *P*-value < 0.05) interactions over a background model using MaxHiC [135]. Finally, a count filter (at least 5 sequencing read pairs mapped to the interaction) and a distance filter (distance between two interacting bins must larger than 5 kb) were applied to the identified significant interactions to select Tregs specific chromatin interactions, which were used in the 3DFAACT-SNP workflow.

### RNA-seq data processing

The raw sequencing data were first trimmed using *AdapterRemoval* (ver 2.2.1a) [136] with default parameters to remove sequencing adapters. Trimmed reads were then aligned to hg38 using *STAR* (ver 2.7.0d) [137]. The resulting BAM files were converted into bedgraph files using *bamCoverage* from *deepTools* [138] with count normalised using counts per million mapped reads (CPM).

### Topologically associated domain identification

Hi-C interactions of Tregs were mapped to equal-size bins (20 kb) of the hg38 genome and normalised using *ICE* [32], resulting in a normalised interaction matrix. The matrix was then used as input to identify topologically associated domains (TADs) via *TopDom* [139].

### Visualisation and downstream analyses

Gene set enrichment analysis (GSEA) was performed using a function *enrichr* from the R package *clusterProfiler* [140] with the hallmark gene sets and C5 gene sets (gene ontology terms) from Molecular Signatures Database (MSigDB). Adjusted *P*-value (Benjamini–Hochberg adjusted) of 0.05 is set as the significant threshold. Visualisation of normalised Hi-C interaction matrices (Figs. 2, 3, and 5, Additional file1: Fig. S4–S12) was performed on 40 kb resolution using an in-house R function *hicHeatmap*. The visualisations of individual filtered T1D-associated SNP loci (Figs. 2, 3, 5 and Additional file 1: Fig. S4–S13) were constructed using the R packages *Gviz* [141], *GenomicInteractions* [142] and *coMET* [143].

## Supplementary Information

**Additional file 1.** Figures. S1–S14, and Tables S1, S2.

**Additional file 2.** Promoters and enhancers used in the 3DFAACTS-SNP workflow in this study.

**Additional file 3.** Table S3–S10.

**Additional file 4.** Transcription factor footprint identified from rest and stimulated Tregs ATAC-seq data using HINT-ATAC.

## Data Availability

The Treg ATAC-seq and Hi-C datasets analysed during the current study are available in the European Nucleotide Archive (ENA) repository (PRJEB39882). Published data: FOXP3 ChIP-chip data used during the current study are available on Gene Expression Omnibus (accession no. GSE20995) [31]. Treg and Th1 RNA-seq data used during the current study are available on European Nucleotide Archive (accession no. ERR1198158 and ERR1198159) [41]. Database used in the current study: NIH Roadmap Epigenomics Project: http://www.roadmapepigenomics.org/ [30]. SEdb: http://www.licpathway.net/sedb/ [39]. DICE: https://dice-database.org/landing [42]. gnomAD: https://gnomad.broadinstitute.org/ [86]. FANTOM5: https://fantom.gsc.riken.jp/5/ [29]. Source code for 3DFAACTS-SNP workflow and related in-house scripts are available in GitHub (https://github.com/ningbioinfostruggling/3DFAACTS-SNP).

## Abbreviations

GWAS: Genome-wide association study
SNP: Single nucleotide polymorphism
T1D: Type 1 diabetes
Treg: Regulatory T cells
Tconv: Conventional T cells
FOXP3: Foxhead box protein 3
TF: Transcription factor
eQTL: Expression quantitative trait loci
ChIP-seq: Chromatin immunoprecipitation sequencing
ATAC-seq: Assay for transposase-accessible chromatin sequencing
Hi-C: High-resolution chromosome conformation capture sequencing
TAD: Topologically associated domain
SE: Super-enhancer
