# Supplementary material for: 3DFAACTS-SNP: using regulatory T cell-specific epigenomics data to uncover candidate mechanisms of type 1 diabetes (T1D) risk
Source: Epigenetics Chromatin. 2022 Jun 30;15:24. doi: 10.1186/s13072-022-00456-5 (PMC9244893; doi:10.1186/s13072-022-00456-5)
Supplement: Supplementary file 1 — Additional file 1. Figures. S1–S14, and Tables S1, S2. [file 13072_2022_456_MOESM1_ESM.docx]

***Supplementary information***

3DFAACTS-SNP: Using regulatory T cell-specific epigenomics data to uncover candidate mechanisms of Type-1 Diabetes (T1D) risk

Ning Liu^1,2,3,+^, Timothy Sadlon^2,4,+^, Ying Ying Wong^2,4^, Stephen Pederson^3^, James Breen^1,2,3,*^ & Simon C Barry^2,4^

^1^ South Australian Health & Medical Research Institute, Adelaide, Australia

^2^ Robinson Research Institute, University of Adelaide, Adelaide, Australia

^3^ Bioinformatics Hub, School of Biological Sciences, University of Adelaide, Adelaide, Australia

^4^ Womens & Childrens Hospital, Adelaide, Australia

^+^ These authors contributed equally

* Corresponding authors: James Breen ([jimmy.breen@sahmri.com](mailto:jimmy.breen@sahmri.com))

**Supplementary Tables**

**Table S1.** Statistics of Tregs ATAC-seq data.

| Metrics | Rest Treg D1 | Stim Treg D1 | Rest Treg D2 | Stim Treg D2 | Rest Treg D3 | Stim Treg D3 |
| --- | --- | --- | --- | --- | --- | --- |
| Raw reads | 41,157,156 | 42,588,662 | 32,775,125 | 38,930,881 | 34,870,964 | 32,230,708 |
| Mapped reads | 36,592,827 (88.91%) | 39,454,136 (92.64%) | 29,327,182 (89.48%) | 35,092,296 (90.14%) | 31,307,151 (89.78%) | 29,726,382 (92.23%) |
| Uniquely mapped | 27,970,773 (67.96%) | 32,704,650 (76.79%) | 22,653,938 (69.12%) | 28,629,417 (73.54%) | 24,474,978 (70.19%) | 24,870,158 (77.16%) |
| Duplication | 3.90% | 6.60% | 4.20% | 6.70% | 4% | 7.20% |
| TSS enrichment score | 8.13 | 23.25 | 16.78 | 10.47 | 12.14 | 25.7 |
| FRiP score | 0.37 | 0.57 | 0.41 | 0.57 | 0.43 | 0.69 |
| Peaks (P-value < 0.05) | 599,103 | 349,878 | 802,822 | 371,943 | 1,003,750 | 339,701 |

**Table S2.** Statistics of Tregs Hi-C data.

| **Metric** | **Count** |
| --- | --- |
| Sequenced Read Pairs | 1,316,463,179 |
| Mapped Read Pairs | 1,301,573,178 |
| Mapped Read Pairs (MAPQ > 30) | 930,858,633 |
| Hi-C Interaction | 661,676,809 |
| Inter Chromosomal | 512,567,849 |
| Intra Chromosomal | 149,108,960 |
| Intra Long Range (> 20kb) | 337,033,192 |
| Statistically significant interactions (5kb resolution, distance > 5kb, read pair count >= 5) | 345,258 |

**Additional File 2**: Promoters and enhancers used in the 3DFAACTS-SNP workflow in this study.

**Table S3**: T1D 3DFAACTS SNPs identified using 3DFAACTS-SNP workflow from T1D fine-mapped SNPs and their 3D interacting genes.

Table S3 is shown in a separate file (Additional File 3).

**Table S4**: Information of the interacting genes of T1D 3DFAACTS SNPs.

Table S4 is shown in a separate file (Additional File 3).

**Table S5**: Topologically-associated domains (TADs) identified using TopDom from Treg Hi-C data (20kb resolution).

Table S5 is shown in a separate file (Additional File 3).

**Additional File 4**: Transcription factor footprint identified from rest and stimulated Tregs ATAC-seq data using HINT-ATAC.

**Table S6-8**: T1D 3DFAACTS SNPs that are located in active transcription factor footprint in Tregs. And the binding affinity effect of these SNPs with their overlapped transcription factor calculated using GWAS4D.

Table S6-8 is shown in a separate file (Additional File 3).

**Table S9**: 3DFAACTS SNPs identified from fine-mapped and meta-analysis SNP datasets from 3 published studies.

Table S9 is shown in a separate file (Additional File 3).

**Table S10**: 3DFAACTS SNPs identified from gnomAD common (MAF >= 0.1) SNPs.

Table S10 is shown in a separate file (Additional File 3).

**Supplementary Figures**


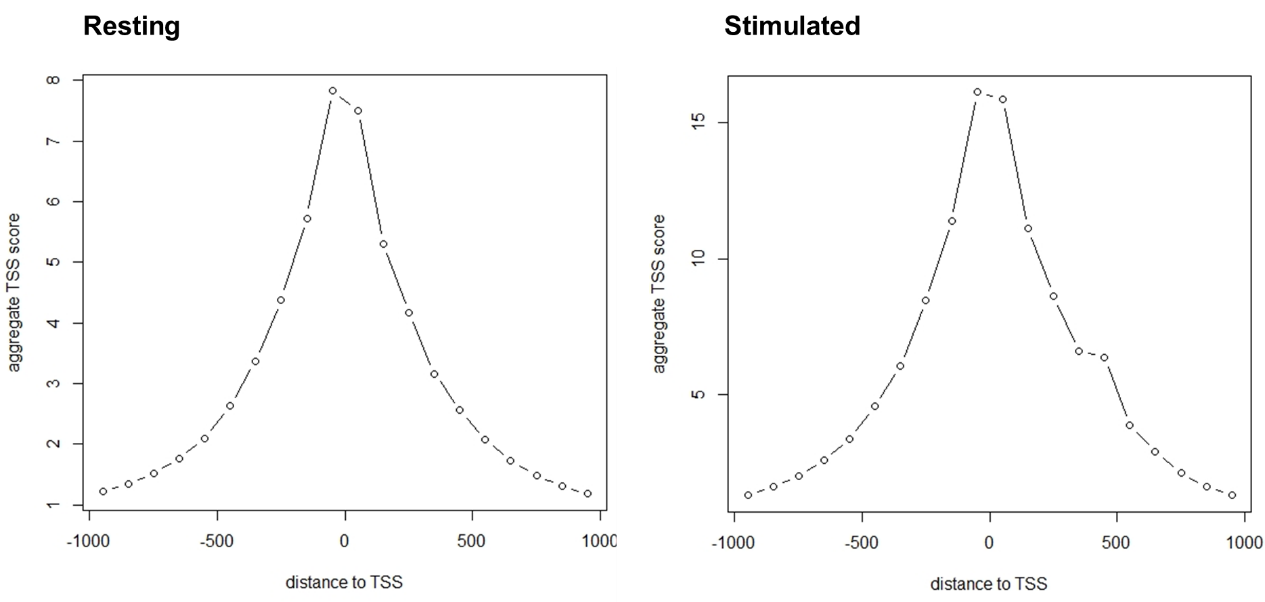


**Figure S1: Enrichment of reads at TSS in data derived from resting (left) and stimulated (right) Treg cells. Each plot represents data from 3 replicates.**


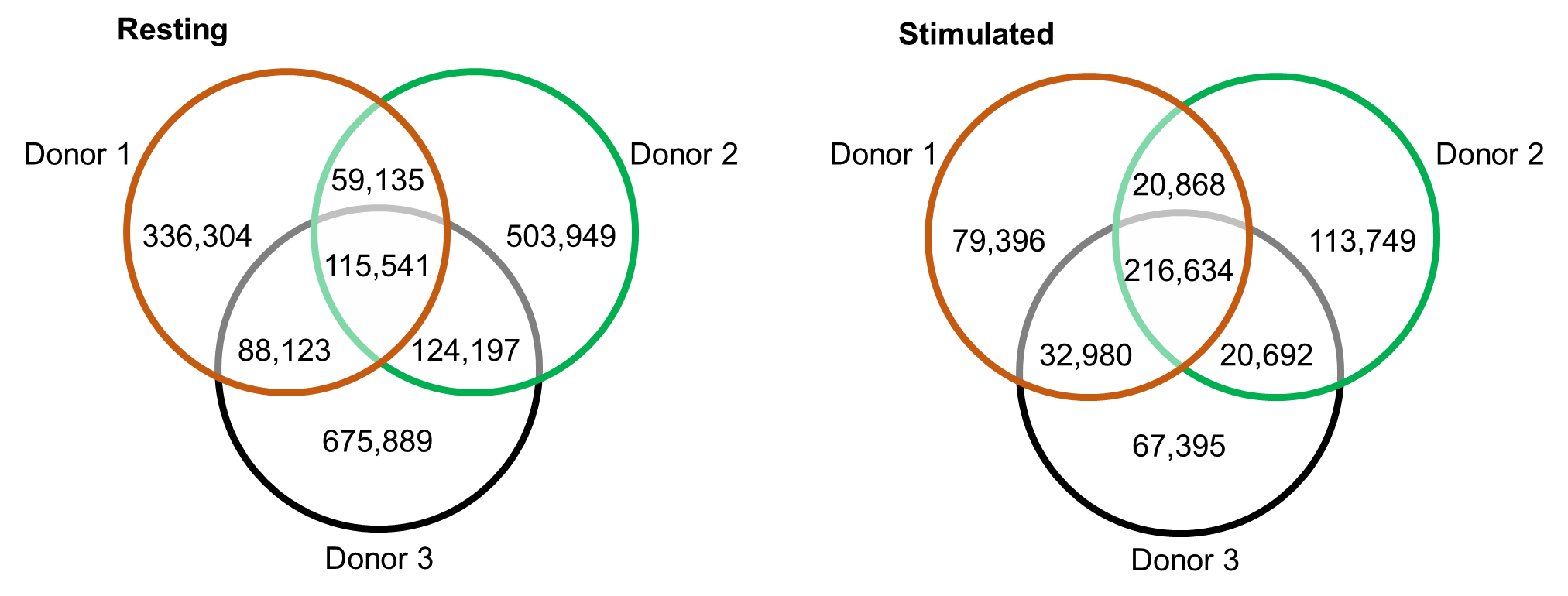


**Figure S2: Overlap of peaks called from all available reads from 3 donors in resting (left) and stimulated (right) conditions.**


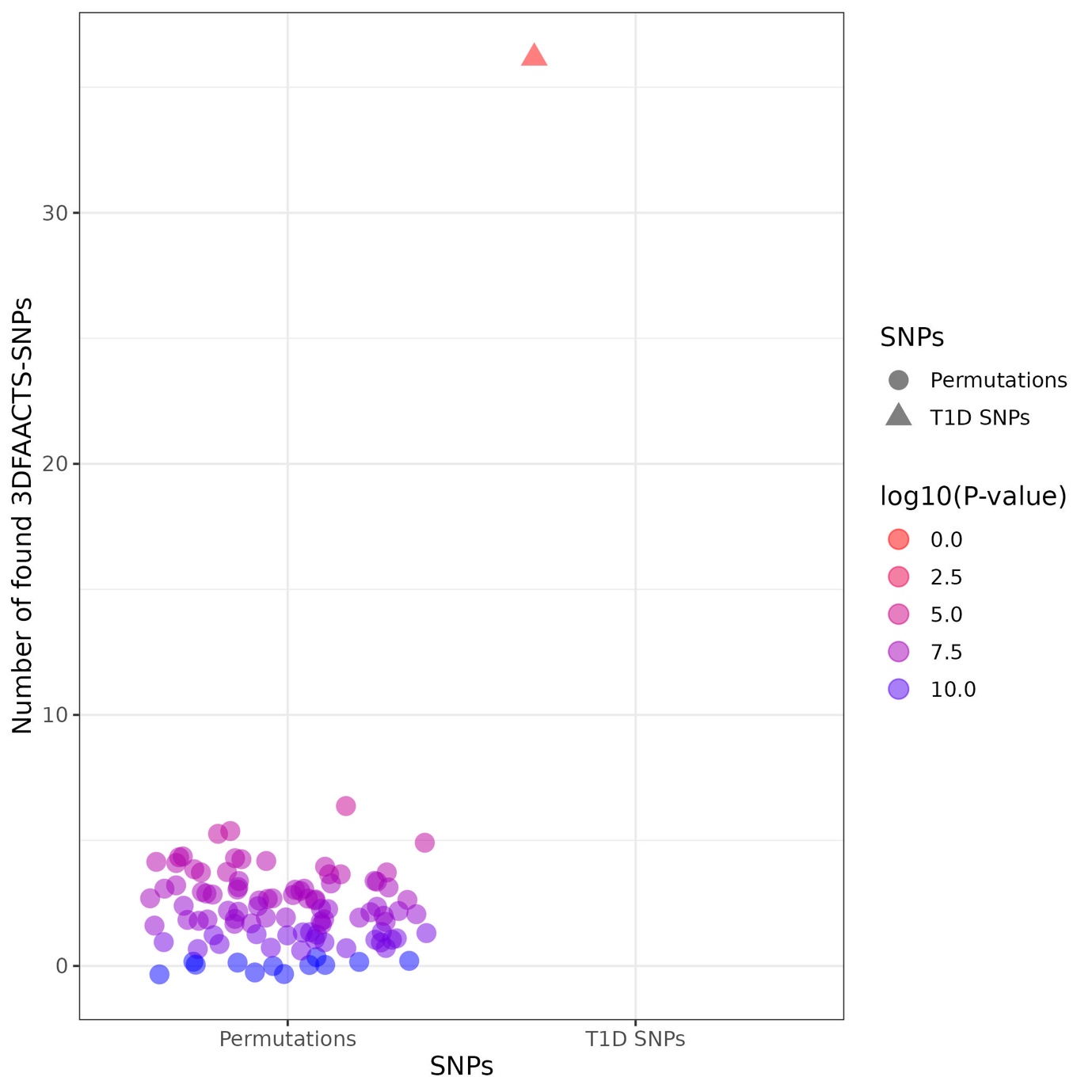


**Figure S3: Comparison between T1D 3DFAACTS SNPs and permuted 3DFAACTS SNPs.**

Scatter plot showing the number of permuted SNPs, which are identified from the 3DFAACTS-SNP workflow with 1228 random genomic loci, from 100 permutations (left) and the number of T1D 3DFAACTS SNPs identified from 1228 T1D SNPs (right).

**Figure S4:** **Visualisation of the region of T1D 3DFAACTS SNPs on chr5:** **35352209-** **36352209.** Tracks displayed below the chromosome 5 ideogram display workflow datasets (filtered SNPs, FOXP3-binding sites and Treg ATAC-seq, statistically significant Hi-C interactions of Tregs (5kb resolution), promoters and enhancers) along with various types of cell type-specific data including UCSC Gene Transcript information, T cell subsets (Thelper1 and Treg) expression data, super-enhancer data, 15-state ChromHMM track of T cell lineages and the heatmap showing the Tregs Hi-C interaction matrix (20kb resolution). For better visualisation, the plotted region of the tracks (chr5: 35352209- 36352209) are the green area in the heatmap. The red triangles indicate the Topologically Associated Domains (TADs).

**Figure S5:** **Visualisation of the region of T1D 3DFAACTS SNPs on chr7:** **49894800-** **50894800.** Tracks displayed below the chromosome 7 ideogram display workflow datasets (filtered SNPs, FOXP3-binding sites and Treg ATAC-seq, statistically significant Hi-C interactions of Tregs (5kb resolution), promoters and enhancers) along with various types of cell type-specific data including UCSC Gene Transcript information, T cell subsets (Thelper1 and Treg) expression data, super-enhancer data, 15-state ChromHMM track of T cell lineages and the heatmap showing the Tregs Hi-C interaction matrix (20kb resolution). For better visualisation, the plotted region of the tracks (chr7: 49894800- 50894800) are the green area in the heatmap. The red triangles indicate the Topologically Associated Domains (TADs).

**Figure S6:** **Visualisation of the region of T1D 3DFAACTS SNPs on chr10:** **5552734-** **6552734.** Tracks displayed below the chromosome 10 ideogram display workflow datasets (filtered SNPs, FOXP3-binding sites and Treg ATAC-seq, statistically significant Hi-C interactions of Tregs (5kb resolution), promoters and enhancers) along with various types of cell type-specific data including UCSC Gene Transcript information, T cell subsets (Thelper1 and Treg) expression data, super-enhancer data, 15-state ChromHMM track of T cell lineages and the heatmap showing the Tregs Hi-C interaction matrix (20kb resolution). For better visualisation, the plotted region of the tracks (chr10: 5552734- 6552734) are the green area in the heatmap. The red triangles indicate the Topologically Associated Domains (TADs).

**Figure S7:** **Visualisation of the region of T1D 3DFAACTS SNPs on chr12:** **9273162-** **10273162.** Tracks displayed below the chromosome 12 ideogram display workflow datasets (filtered SNPs, FOXP3-binding sites and Treg ATAC-seq, statistically significant Hi-C interactions of Tregs (5kb resolution), promoters and enhancers) along with various types of cell type-specific data including UCSC Gene Transcript information, T cell subsets (Thelper1 and Treg) expression data, super-enhancer data, 15-state ChromHMM track of T cell lineages and the heatmap showing the Tregs Hi-C interaction matrix (20kb resolution). For better visualisation, the plotted region of the tracks (chr12: 9273162- 10273162) are the green area in the heatmap. The red triangles indicate the Topologically Associated Domains (TADs).

**Figure S8:** **Visualisation of the region of T1D 3DFAACTS SNPs on chr15:** **38111471-** **39111471.** Tracks displayed below the chromosome 15 ideogram display workflow datasets (filtered SNPs, FOXP3-binding sites and Treg ATAC-seq, statistically significant Hi-C interactions of Tregs (5kb resolution), promoters and enhancers) along with various types of cell type-specific data including UCSC Gene Transcript information, T cell subsets (Thelper1 and Treg) expression data, super-enhancer data, 15-state ChromHMM track of T cell lineages and the heatmap showing the Tregs Hi-C interaction matrix (20kb resolution). For better visualisation, the plotted region of the tracks (chr15: 38111471- 39111471) are the green area in the heatmap. The red triangles indicate the Topologically Associated Domains (TADs).

**Figure S9:** **Visualisation of the region of T1D 3DFAACTS SNPs on chr16:** **10595092-** **11595092.** Tracks displayed below the chromosome 16 ideogram display workflow datasets (filtered SNPs, FOXP3-binding sites and Treg ATAC-seq, statistically significant Hi-C interactions of Tregs (5kb resolution), promoters and enhancers) along with various types of cell type-specific data including UCSC Gene Transcript information, T cell subsets (Thelper1 and Treg) expression data, super-enhancer data, 15-state ChromHMM track of T cell lineages and the heatmap showing the Tregs Hi-C interaction matrix (20kb resolution). For better visualisation, the plotted region of the tracks (chr16: 10595092- 11595092) are the green area in the heatmap. The red triangles indicate the Topologically Associated Domains (TADs).

**Figure S10:** **Visualisation of the region of T1D 3DFAACTS SNPs on chr17:** **40099413-** **41099413.** Tracks displayed below the chromosome 17 ideogram display workflow datasets (filtered SNPs, FOXP3-binding sites and Treg ATAC-seq, statistically significant Hi-C interactions of Tregs (5kb resolution), promoters and enhancers) along with various types of cell type-specific data including UCSC Gene Transcript information, T cell subsets (Thelper1 and Treg) expression data, super-enhancer data, 15-state ChromHMM track of T cell lineages and the heatmap showing the Tregs Hi-C interaction matrix (20kb resolution). For better visualisation, the plotted region of the tracks (chr17: 40099413- 41099413) are the green area in the heatmap. The red triangles indicate the Topologically Associated Domains (TADs).

**Figure S11:** **Visualisation of the region of T1D 3DFAACTS SNPs on chr18:** **12338768-** **13338768.** Tracks displayed below the chromosome 18 ideogram display workflow datasets (filtered SNPs, FOXP3-binding sites and Treg ATAC-seq, statistically significant Hi-C interactions of Tregs (5kb resolution), promoters and enhancers) along with various types of cell type-specific data including UCSC Gene Transcript information, T cell subsets (Thelper1 and Treg) expression data, super-enhancer data, 15-state ChromHMM track of T cell lineages and the heatmap showing the Tregs Hi-C interaction matrix (20kb resolution). For better visualisation, the plotted region of the tracks (chr18: 12338768- 13338768) are the green area in the heatmap. The red triangles indicate the Topologically Associated Domains (TADs).

**Figure S12:** **Visualisation of the region of T1D 3DFAACTS SNPs on chr22:** **29685733-** **30685733.** Tracks displayed below the chromosome 22 ideogram display workflow datasets (filtered SNPs, FOXP3-binding sites and Treg ATAC-seq, statistically significant Hi-C interactions of Tregs (5kb resolution), promoters and enhancers) along with various types of cell type-specific data including UCSC Gene Transcript information, T cell subsets (Thelper1 and Treg) expression data, super-enhancer data, 15-state ChromHMM track of T cell lineages and the heatmap showing the Tregs Hi-C interaction matrix (20kb resolution). For better visualisation, the plotted region of the tracks (chr22: 29685733- 30685733) are the green area in the heatmap. The red triangles indicate the Topologically Associated Domains (TADs).

**Figure S13:** **Published ChIA-PET data displaying the differences of interaction count after TNF induction in the CCR2/3/5 loci on chr3:45600000-46840000.**

**Figure S14**: **Enrichment of gnomAD 3DFAACTS SNPs found within NIH Epigenomics Roadmap samples.** Enrichment test of filtered gnomAD SNPs against chromHMM states from 129 tissues and cell types from Epigenomics Roadmap using GIGGLE. Red coloured regions indicate positive enrichment of variants within cell-types and chromHMM states, while blue coloured regions indicate negative enrichment.

**Figure S15**: **Enrichment of 3D interacting regions of gnomAD 3DFAACTS SNPs found within NIH Epigenomics Roadmap samples.** Enrichment test of filtered gnomAD SNPs against chromHMM states from 129 tissues and cell types from Epigenomics Roadmap using GIGGLE. Red coloured regions indicate positive enrichment of variants within cell-types and chromHMM states, while blue coloured regions indicate negative enrichment.

**Figure S16: Top20 significantly enriched gene sets of common gnomAD 3DFAACTS SNPs 3D interacting genes.**
